# Compulsory Vaccination

**Published:** 1898-07-30

**Authors:** 


### COMPULSORY VACCINATION.

In view of the tender-heartedness towards the conscientious objector which is displayed by many Members of Parliament, and curiously enough even by the representatives of medicine, it is not without interest to observe what are the real opinions of some who still are proposing to vote in favour of amendments which will have the effect of making compulsion of no effect. Sir Robert Farquharson, for example, addressing the annual meeting of the Metropolitan Counties Branch of the British Medical Association, said "No one has a greater objection than I have to the conscientious objector, for he is usually a feather-headed person, stuffed with vague abstract views about personal liberty and State interference, and falling an easy prey to the sophistical arguments of the paid antivaccination official. I should like, in the interest of the helpless rising generation, to punish the dogmatic parent severely and repeatedly, and make his life a burden till he obeys the law." This is common sense, and in all that he says we are sure that he will receive the support of the medical profession. Yet he proposes to vote with Sir Walter Foster.

